# Involvement of the Endocrine-Disrupting Chemical Bisphenol A (BPA) in Human Placentation

**DOI:** 10.3390/jcm9020405

**Published:** 2020-02-03

**Authors:** Sophie-Christine de Aguiar Greca, Ioannis Kyrou, Ryan Pink, Harpal Randeva, Dimitris Grammatopoulos, Elisabete Silva, Emmanouil Karteris

**Affiliations:** 1 College of Health and Life Sciences, Brunel University London, Uxbridge UB8 3PH, UK; sophieja3@gmail.com; 2 Aston Medical Research Institute, Aston Medical School, Aston University, Birmingham B4 7ET, UK; i.kyrou@aston.ac.uk; 3 Warwickshire Institute for the Study of Diabetes, Endocrinology and Metabolism (WISDEM), University Hospitals Coventry and Warwickshire NHS Trust, Coventry CV2 2DX, UK; 4 Institute of Precision Diagnostics and Translational Medicine, UHCW NHS Trust, Coventry CV4 7AL, UK; harpal.randeva@warwick.ac.uk (H.R.); dimitris.grammatopoulos@warwick.ac.uk (D.G.); 5 Warwick Medical School, University of Warwick, Coventry CV4 7AL, UK; 6 Dept of Bio. & Med. Sci., Oxford Brookes University, Oxford OX3 0BP, UK; r.pink@brookes.ac.uk

**Keywords:** endocrine-disrupting chemicals, BPA, placenta, microarray

## Abstract

Background: Endocrine-disrupting chemicals (EDCs) are environmental chemicals/toxicants that humans are exposed to, interfering with the action of multiple hormones. Bisphenol A (BPA) is classified as an EDC with xenoestrogenic activity with potentially adverse effects in reproduction. Currently, a significant knowledge gap remains regarding the complete spectrum of BPA-induced effects on the human placenta. As such, the present study examined the effects of physiologically relevant doses of BPA in vitro. Methods: qRT-PCR, Western blotting, immunofluorescence, ELISA, microarray analyses, and bioinformatics have been employed to study the effects of BPA using nonsyncytialised (non-ST) and syncytialised (ST) BeWo cells. Results: Treatment with 3 nM BPA led to an increase in cell number and altered the phosphorylation status of p38, an effect mediated primarily via the membrane-bound estrogen receptor (GPR30). Nonbiased microarray analysis identified 1195 and 477 genes that were differentially regulated in non-ST BeWo cells, whereas in ST BeWo cells, 309 and 158 genes had altered expression when treated with 3 and 10 nM, respectively. Enriched pathway analyses in non-ST BeWo identified a leptin and insulin overlap (3 nM), methylation pathways (10 nM), and differentiation of white and brown adipocytes (common). In the ST model, most significantly enriched were the nuclear factor erythroid 2-related factor 2 (NRF2) pathway (3 nM) and mir-124 predicted interactions with cell cycle and differentiation (10 nM). Conclusion: Collectively, our data offer a new insight regarding BPA effects at the placental level, and provide a potential link with metabolic changes that can have an impact on the developing fetus.

## 1. Introduction

Endocrine-disrupting chemicals (EDCs) are environmental chemicals (e.g., chemicals in manufacturing and packaging materials) with the potential of disrupting the endocrine system of humans and wildlife [1]. To date, among the hundreds of thousands of synthetic chemicals, several hundred have been recognized as potentially having endocrine active properties [2]. EDCs are widespread in the environment and can accumulate throughout the food chain, particularly since most of these lipophilic chemicals exhibit long half-lives [3]. A large body of research has indicated that in humans, prolonged exposure to these chemicals can be associated with metabolic dysfunction, disorders of the reproductive system, endocrine-related cancers, and neurodevelopmental diseases [4,5,6,7,8,9,10,11].

There are numerous groups of EDCs with very diverse uses, including plasticisers (e.g., bisphenol A, BPA; and phthalates), pesticides (e.g., dichlorodiphenyltrichloroethane, DDT; and dieldrin), flame retardants (e.g., polybrominated diphenyl ethers), and additives to consumer goods (e.g., parabens, benzophenone, and synthetic musks such as galaxolide) [1]. BPA is an EDC which was first synthesised in 1891 [12] and is now widely used in a variety of products (e.g., plastics, lining of aluminium cans, and thermal receipts) [13], thus representing one of the most frequently detected emerging pollutants in the environment [14]. Structurally, BPA consists of a phenolic and hydroxyl group bound to an aromatic ring [BPA chemical formula: (CH_3_)_2_C(C_6_H_4_OH)_2_], which can bind to other compounds to form polymers when used in manufacturing [14,15,16]. Certain conditions, such as heat and acidic or basic environments, can cause leaching of BPA to its surroundings, leading to potential environmental and human (predominantly oral) exposure to BPA [17]. This exposure appears to be linked to a number of health risks, since BPA interacts with nuclear estrogen receptors (ERα and ERβ), membrane-bound estrogen receptors (e.g., GPR30), and other receptors (e.g., human nuclear receptor estrogen-related receptor γ) [18,19,20].

In humans, BPA has been detected in fetal and maternal plasma, as well as in amniotic and follicular fluid, whilst deposits have also been found in placenta tissue [21]. Variable BPA concentrations have been measured in these compartments in humans, ranging from 0.3 to 18.9 ng/mL and 0.2 to 9.2 ng/mL in maternal and fetal plasma, respectively, as well as 1.0 to 104.9 ng/g in term placenta [21,22]. An increasing body of evidence has shown that BPA has neurobehavioural, neurotoxic, and neuroendocrine effects. The impact of BPA on neurodevelopment is not only linked to the effect on the placenta. There is also evidence of a direct effect on the fetus, such as the HPA axis, thyroid receptors, and estrogen receptors [9,23,24,25]. Moreover, BPA exposure in utero appears to be associated with implantation problems, as well as preeclampsia, preterm births, and low birth weight [26,27,28,29,30]. Indeed, fetal malformation was shown to be higher in offspring from mothers with higher levels of free circulating BPA levels [31], whilst there is a potential association between BPA exposure and low birth weight of infants, especially female [27]. Furthermore, high doses of BPA have been shown to affect the growth of offspring in the first years of life [31,32]. Work conducted with concentrations found in human tissues can also induce behavioural and neuronal alterations and cognitive deficits [31].

Understanding the effects of environmental chemicals during gestation is crucial, as normal fetal development paves the way for subsequent normal development and growth [33]. We therefore hypothesized that BPA can affect placentation and subsequently gestation by activating placental estrogen receptors. Several studies have shown that BPA can exert proliferative effects acting in a genomic and nongenomic manner in vitro and activate signalling cascades such as Akt and MAPK [25,34,35,36,37,38].

In this study we have used placental cells (BeWo) as an in vitro model to study the effects of high and low physiologically relevant concentrations of BPA. We have investigated initially the effect of BPA in nonsyncytialised BeWos as a marker of 1st trimester trophoblasts by measuring changes in cell number, activation of signalling cascades, and impact on gene expression using microarrays followed by validation of these mRNA changes. We then expanded on our observations by assessing the effects of BPA in a syncytialised model of BeWo cells, therefore resembling the endocrine-active component of the human placenta. To the best of our knowledge, this is the first time that the effect of BPA in two different states of placentation has been assessed.

## 2. Materials and Methods

### 2.1. Cell Culture

BeWo (CCL-98™ ATCC^®^, Teddington, UK; is a human choriocarcinoma-derived cell line with human trophoblastic qualities able to cellularly differentiate in vitro to syncytiotrophoblast cells using 8-bromo-cAMP or forskolin [39,40,41]. BeWo cells were grown in Ham F12 Medium supplemented with 10% fetal bovine serum (FBS) (Gibco™, Thermo Fisher Scientific, Waltham, MA USA) and 0.1% of penicillin/streptomycin. The cells were maintained in 75 cm^2^ nontreated culture flasks (Thermo Fisher Scientific, Waltham, MA USA) under standard culture conditions in a humidified atmosphere containing 5% CO_2_ at 37 °C.

### 2.2. Syncytialisation Using 8-Bromo-cAMP and Treatment with Bisphenol A (BPA)

BeWo cells were seeded onto 6-well plates and left to grow for 24 h with 2 mL of media and treated with 50 μM 8-Bromo-cAMP (Tocris Bioscience™, Abington, UK) dissolved in sterile H_2_O for 72 h (estimated confluence at treatment ~70%). For hormone treatments, cells were plated on 6-well plates and incubated at standard culture conditions. After 24 h, media was changed and cells were treated for 24 h with 3 nM (a physiologically relevant concentration) BPA (Sigma-Aldrich, Gillingham, UK) dissolved in ethanol, 10 nM BPA, 30 nM β-estradiol (E2; Sigma-Aldrich, Gillingham, UK), or pure ethanol as a control. After 24 h, cells were processed for further experiments.

### 2.3. RNA Extraction, cDNA Synthesis, and Quantitative Reverse Transcription PCR (qRT-PCR)

Cells were grown in 6-well plates and treated as mentioned above. RNA was extracted using the GenElute Mammalian Total RNA miniprep kit (Sigma-Aldrich, Gillingham, UK), following the manufacturer’s instructions. Every sample was measured using the NanoDrop 2000C (Thermo Fisher Scientific, Waltham, MA USA) spectrophotometer. Concentration was assessed by A260/A280 ratio, a range of 1.7–2.0 classified as acceptable. cDNA synthesis was performed using the Precision NanoScript™ 2 Reverse Transcription Kit (Primerdesign, Camberley, UK) according to the manufacturer’s instructions. PrecisionPlus mastermix premixed with SYBR green (Primerdesign, Camberley, UK) and primers as described in Table 1 were used for qPCR. A QuantStudio™ 7 Flex System Real-Time PCR System machine (Applied Biosystems, CA, USA) was used for the study.

Two-colour microarray-based gene expression using a low input Quick Amp labelling kit was measured using Agilent Gene Expression SurePrint G3 Human GE v2 8x60k oligo microarrays using a Sure Scan microarray scanner (Agilent, CA, USA). RNA was extracted from samples treated with 3 nM and 10 nM BPA, as previously described, and 100 ng of RNA as input was used per sample. Scanning and feature extraction were performed on a SureScan microarray scanner (Agilent, CA, USA) using Feature Extraction software v12.0.

FunRich v3.1.3 [42] and Enrichr [43] were used for further analyses. FunRich analyses biological processes, cellular components, protein domains and molecular functions, expression sites, biological pathways, and transcription factors and provides a clinical synopsis of phenotypic terms using many of the common genomic databases. To further analyse data, differentially expressed gene lists were uploaded to the online bioinformatics application Enrichr. Enrichr uses databases, such as NCI Nature and Go Molecular Function, to assess gene enrichment in terms of molecular function, biological processes, biological pathways, transcription factors, diseases, and other gene enrichment groups.

### 2.4. Western Blotting

Protein lysates were extracted from transfected and control BeWo cells. Proteins were first separated by a 10% v/v SDS-PAGE and then the separated proteins were electrophoretically transferred onto a nitrocellulose membrane (Thermo Scientific, Waltham, MA USA). After wet transfer, membranes were blocked in 5% milk powder in 1x TBS Tween for one hour. Membranes were treated with primary antibodies for AKT, p38, ERK1/2 (total and phospho), and GAPDH (Cell Signaling Technology^®^, MA, USA) in 5% BSA at a dilution of 1:1000 and incubated at 4 ℃ overnight. Membranes were washed 3 times for 15 min with 1x TBS Tween. Secondary anti-rabbit HRP-conjugated secondary antibody (Cell Signaling Technology^®^, MA, USA) diluted in 5% BSA at a dilution of 1:2000 was added, and membranes were incubated at room temperature for 1 h. Membranes were washed 3 times for 15 min with 1x TBS Tween followed by developing, as previously described [44].

### 2.5. Immunofluorescence

BeWo cells were grown and seeded at a specific density in a 6-well plate that contained coverslips as above. After 24 h, media was changed to 0.5% FBS starved media and left to incubate for one hour. Then, cells were treated with BPA (3 nM, 10 nM) and E2 (30 nM) for 24 h, 1 h, 30 min, 15 min, and 5 min. Cells were fixed with cold 4% PFA as mentioned above. Cells were stained as previously mentioned using appropriate antibodies for E-Cadherin (Cell Signaling Technology^®^, MA, USA), ERs, and GPR30 (Santa Cruz, Dallas, TX, USA) [44].

### 2.6. Statistical Analysis

Student’s t-test or ANOVA tests were performed on data of equal variance in order to compare data sets. In unequal variance data, the Mann–Whitney U test was used to determine significance. Values were significant at *p* < 0.05 (*), *p* < 0.01 (**), and *p* < 0.001 (***).

## 3. Results

### 3.1. BPA Effects on Phosphorylation of Key Kinases and Cell Number

**Figure 1:**
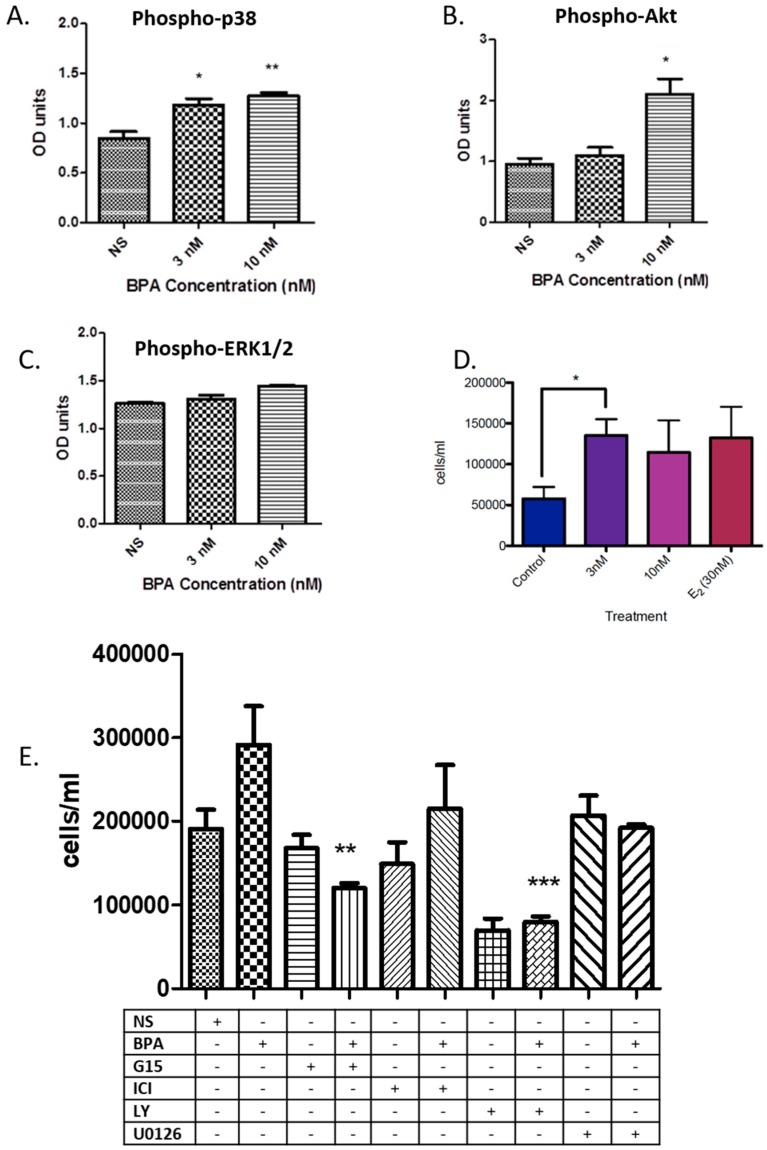
(**A**,**B**). Relative amount of phospho-p38 (A) and phospho-Akt after 60 min of bisphenol A (BPA) treatment (3 nM and 10 nM). Treatment of BeWo cells with 3 nM and 10 nM BPA significantly increased the expression of p-p38 after 60 min (* *p* < 0.05 and ** *p* < 0.01 compared to no supplement (NS)) (A). Treatment of BeWo cells with 10 nM BPA significantly increased the expression of p-AKT after 60 min (* *p* < 0.05 compared to NS) (B). Both protein expression of the housekeeping gene GAPDH and of total p38 remained unchanged; (**C**). There was no difference in the phosphorylation status of ERK1/2 when cells were treated with BPA for 60 min; (**D**). Changes in BeWo cell number treated with 3 nM BPA, 10 nM BPA, and 30 nM estradiol (E2). The 3 nM BPA treatment significantly increased cell number compared to controls (*p* < 0.05), while there was a notable, but not significant, increase in number when cells were treated with 10 nM BPA or 30 nM E2; (**E**). Changes in the number of BeWo cells treated with 3 nM BPA and/or estrogen receptor (ER) antagonists (i.e., ICI 182,780 (ICI): ERα and ERβ inhibitor, G15: GPR30 inhibitor). Cell number of BPA-treated cells was significantly decreased when treated with G15 (*p* < 0.05). There was also a significant decrease in cell number when cells were treated with LY294002 (LY), as well as for the treatment with BPA + LY294002 when compared to controls and treatment with only BPA (*** *p* < 0.001 compared to control). There was a decrease in cell number when cells were treated with U0126 or BPA + U0126 when compared to treatment with only BPA just short of significance (*p* = 0.05).

In order to assess the short-term effect of BPA on undifferentiated (i.e., nonsyncytialised) BeWo cells, these were treated with BPA at physiologically relevant concentrations of 3 nM and 10 nM for 5 to 60 min and phosphorylation levels of p38, ERK1/2, and AKT were measured, since they are known modulators of trophoblast biology. After 60 min of treatment (Figure 1A), phospho-p38 levels were significantly increased in both 3 nM (*p* < 0.05) and 10 nM treated cells (*p* < 0.01). A statistically significant increase by 2-fold in the phosphorylation status of AKT was observed after 60 min following a 10 nM treatment with BPA (*p* < 0.05; Figure 1B). Phospho-ERK1/2 expression remained unaltered at all tested time points after exposure to both 3 nM or 10 nM BPA (Figure 1C). 

BPA at 3 nM for 24 h was also able to significantly increase cell numbers (*p* < 0.05; Figure 1D). We dissected this response further by using the PI3K-inhibitor LY294002 and the MAPK-inhibitor UO126, as well as the estrogen receptor antagonist ICI 182,780 (ERα and ERβ inhibitor) and the GPR30 inhibitor G15 in the presence of BPA. When treating BeWo cells with ER antagonists, there was a significant decrease in cell numbers over 24 h when cells were treated with G15 and a moderate—but not significant—decrease when treated with the ERα antagonist ICI 182,780 (Figure 1E). There was a significant decrease in cell numbers over 24 h when treated with 3 nM BPA in the presence of LY294002 (*p* < 0.001; Figure 1E), and just short of significance for the U0126 (*p* = 0.05; Figure 1E). 

### 3.2. Gene Microarray Analyses Assessing the Effects of BPA in Nonsyncytialised BeWo Cells

BeWo cells were treated with 3 nM or 10 nM BPA for 24 h in order to assess effects of BPA on transcription and cell functions and pathways on nonsyncytialised BeWo cells. Genes were classified from highest to lowest p-value and highest to lowest fold-change compared to untreated controls and analysed using the FunRich bioinformatics analysis software. Microarray analysis identified 1195 genes that were differentially regulated in 3 nM treated nonsyncytialised BeWo cells, whereas the 10 nM treated cells only showed differential regulation of 477 genes. A total of 194 genes were commonly regulated by both concentrations.

Of the top upregulated genes (according to p-values) in 3 nM treated nonsyncytialised BeWo cells, the most significantly upregulated gene was the cytoplasmatic polyadenylation element-binding protein 1 (CPEB1), which is vital for cell cycle progression, particularly prophase entry. Other coregulated genes included Rap guanine nucleotide exchange factor (RAPGEF1), Myosin Light Chain 3 (MYL3), Caveolin-1 (CAV1), Calsyntenin-3 (CLSTN3), Hydroxycarboxylic acid receptor 3 (HCAR3), Serpin B9 (SERPINB9), Alanine–glyoxylate aminotransferase 2 (AGXT2), Transmembrane protein 45B (TMEM45B), and Eukaryotic translation initiation factor 4E type 2 (EIF4E2). Similarly, amongst the most upregulated genes following 10 nM BPA treatment were CAV1, followed by MYL3, Cerebellin-1 (CBLN1), Ankyrin-3 (ANK3), Thiopurine S-methyltransferase (TPMT), Leptin (LEP), Hyaluronan and proteoglycan link protein 3 (HAPLN3), Sperm flagellar 1 (SPEF1), Placenta-specific 8 (PLAC8), and EIF4E2. 

### 3.3. Validation and Enrichment Analyses on Gene Microarrays

**Figure 2:**
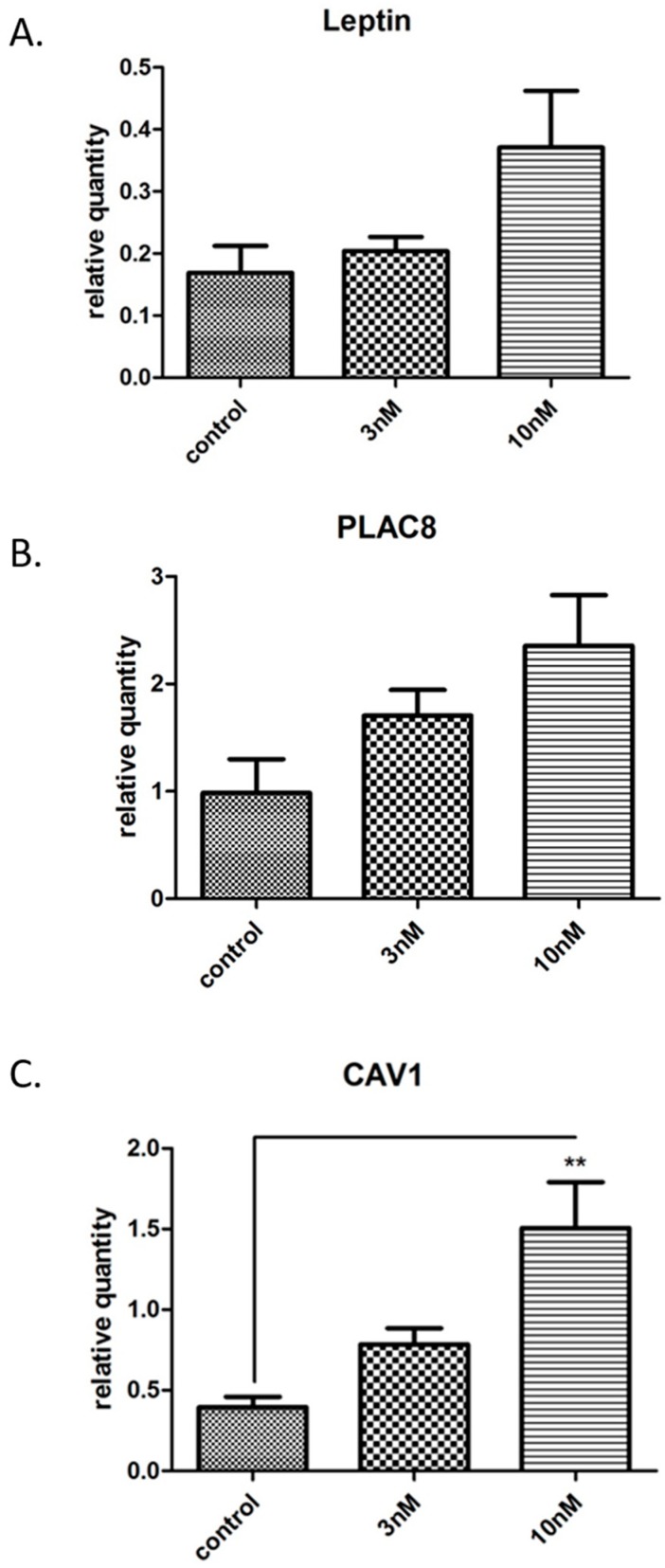
Validation of microarray data using qRT-PCR. (**A**). There was a relative increase in leptin gene expression when compared to controls after 10 nM bisphenol A (BPA) treatment. This is in accordance with the fold-change difference (2.22) between untreated BeWo cells and 10 nM BPA-treated BeWo cells found in microarray analysis; (**B**). There was a notable increase in Placenta-specific 8 (PLAC8) gene expression when compared to controls after 3 nM and 10 nM BPA treatment. This is in accordance with the fold-change difference found between untreated BeWo cells and 3 nM BPA-treated BeWo cells (1.75), as well as between untreated BeWo cells and 10 nM BPA-treated BeWo cells (2.14); (**C**). There was a significant increase in Caveolin-1 (CAV1) when comparing untreated BeWo cells to 10 nM BPA-treated BeWo cells (*p* < 0.01). This is in accordance with the fold-change difference found between untreated BeWo cells and 3 nM BPA-treated BeWo cells (2.35), as well as untreated BeWo cells and 10 nM BPA-treated BeWo cells (3.55).

In order to validate the outcome of the microarray analyses, certain upregulated genes were selected and transcription levels were assessed using qRT-PCR. The genes chosen for validation were CAV1, Leptin, and PLAC8, which were chosen on the basis of possible relation to placental function or cell function. These three genes were significantly upregulated in both 3 nM and 10 nM BPA-treated undifferentiated (nonsyncytialised) BeWo cells. There was a relative increase in leptin gene expression when compared to controls after 10 nM BPA treatment (Figure 2A). This is in accordance with the 2.22-fold change difference between untreated BeWo cells and 10 nM BPA-treated cells seen in microarray analysis. Similarly, there was a relative increase in PLAC8 gene expression when compared to controls. This was also in accordance with the 1.75-fold change difference found between untreated BeWo cells and 3 nM BPA-treated BeWo cells, as well as untreated BeWo cells and 10 nM BPA-treated BeWo cells (Figure 2B). Finally, CAV1 gene expression significantly increased in 10 nM BPA-treated BeWo cells compared to controls (*p* < 0.01), whilst an increase that did not reach statistical significance was also noted in BeWo cells after 3 nM BPA treatment (Figure 2C). This was also in accordance with the 2.35-fold change difference found in microarray analysis between untreated BeWo cells and 3 nM BPA-treated BeWo cells.

Enrichment analyses using Enrichr (WikiPathways 2019 Human database) revealed that the biological pathways most significantly associated with differentially expressed genes in 3 nM and 10 nM BPA-treated BeWo cells are associated with insulin resistance (Table 2) and differentiation of white and brown adipocyte (Table 3), respectively.

**Figure 3:**
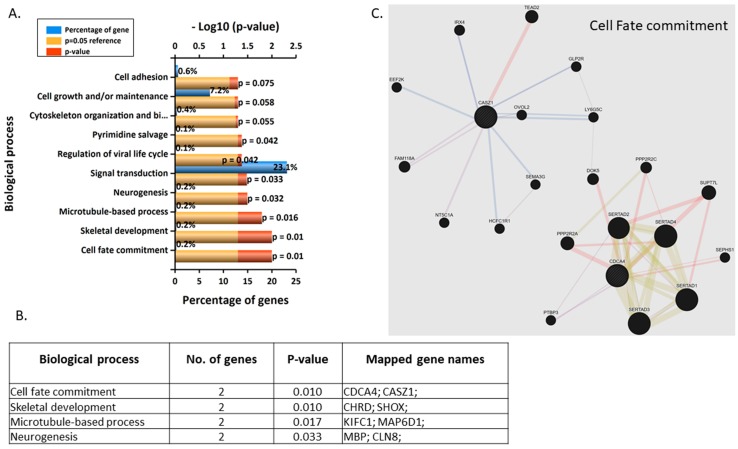
(**A**) Top 10 enriched (Funrich) biological processes for 3 nM BPA-treated BeWos. (**B**) Table of genes involved in biological processes: cell division cycle associated 4 (CDCA4); castor zinc finger 1 (CASZ1); chondrin (CHRD); short stature homeobox (SHOX); kinesin family member C1 (KIFC1); MAP6 domain containing 1 (MAP6D1); myelin basic protein (MBP); ceroid-lipofuscinosis, neuronal 8 (CLN8). (**C**) Network annotation of genes involved in cell fate commitment (Genemania).

The most enriched biological processes were cell fate commitment and skeletal development for 3 nM BPA (Figure 3A,B). The two genes involved in cell fate commitment are CDCA4 and CASZ1, which appear to have two distinct clusters of genes that are associated with (Figure 3C, Supplementary Table S1).

**Figure 4:**
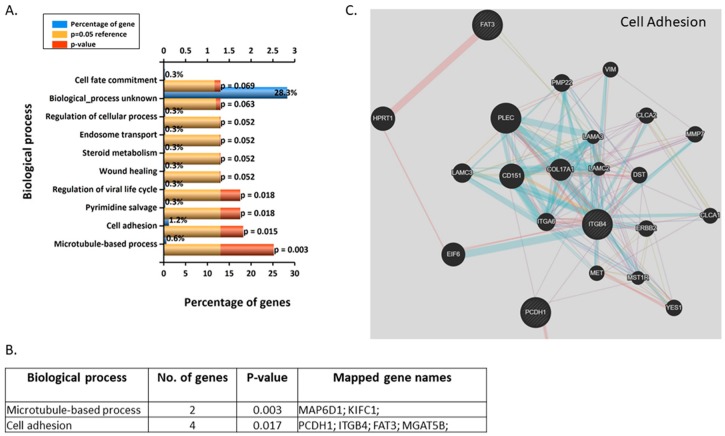
(**A**) Top 10 enriched (Funrich) biological processes for 10 nM BPA-treated BeWos. (**B**) Table of genes involved in biological processes: protocadherin 1 (PCDH1), FAT atypical cadherin 3 (FAT3mannosyl (alpha-1,6-)-glycoprotein beta-1,6-N-acetyl-glucosaminyltransferase, isozyme B (MGAT5B). (**C**) Network annotation of genes involved in cell adhesion (Genemania).

The most enriched biological processes were microtubule-based process, cell adhesion, and pyrimidine salvage for 10 nM BPA (Figure 4A,B). The four genes involved in cell adhesion are PCDH1, ITGB4, FAT3, and MGAT5B, which form an extensive network of genes associated with (Figure 4C, Supplementary Table S2).

### 3.4. Validation of the in Vitro Syncytialisation Model of BeWo Cells

**Figure 5:**
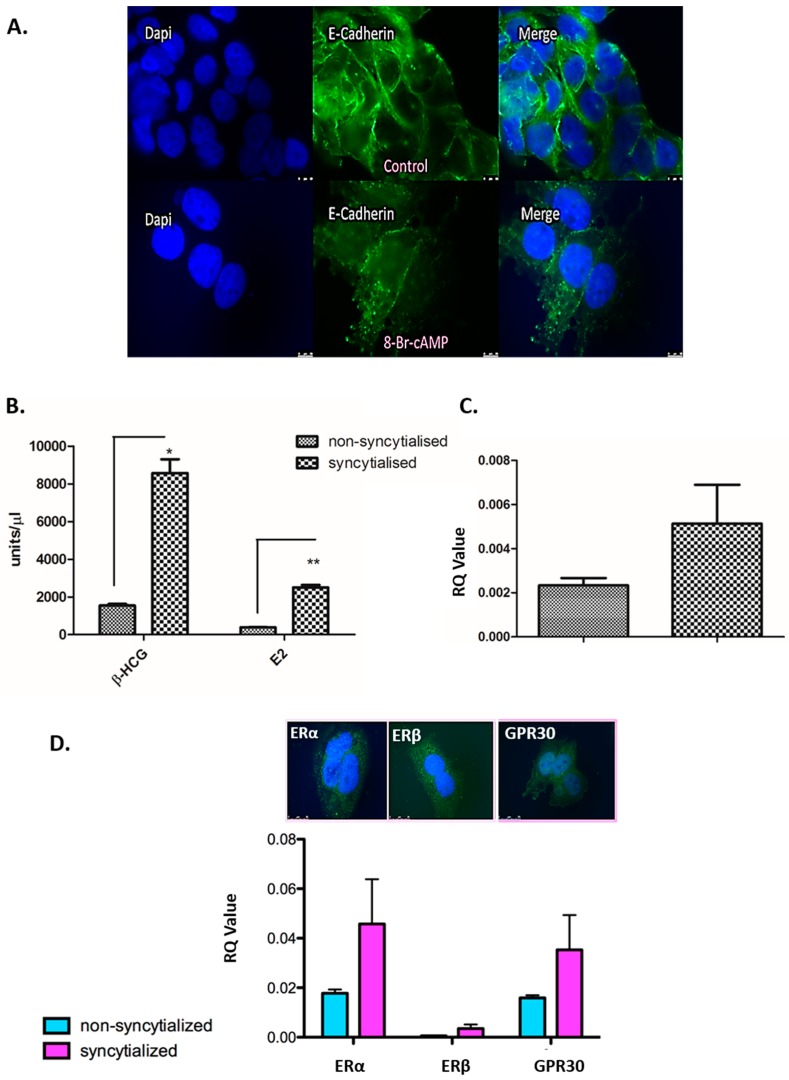
(**A**) Immunofluorescent staining of E-Cadherin (a marker of cell fusion) in BeWo cells treated with 8-bromo-cAMP (8-Br-cAMP) in order to syncytialise. Green: E-Cadherin; blue: DAPI nuclear stain (a blue fluorescent dye used to detect nuclei in fluorescence microscopy). Cells depicted in the bottom row have been treated with 8-Br-cAMP for 72 h, while cells depicted in the top row have not (controls). As BeWo cells treated with 8-Br-cAMP fuse to become syncytia (amorphous and multinucleated cells), cell walls break down and lose E-Cadherin. (**B**) Secretion of estradiol (E2) and β-human chorionic gonadotropin (β-hCG) in conditioned media of nonsyncytialised and syncytialised BeWo cells grown for 24 h. Expression of both β-hCG and E2 was significantly upregulated in syncytialised BeWo cells (*p* = 0.0108 and *p* = 0.0042, respectively) compared to nonsyncytialised BeWo cells. (**C**) Expression of syncytin-2 (a marker of syncytialisation) in nonsyncytialised and syncytialised BeWo cells, showing a more than 2-fold increase in syncytin-2 in the latter (relative quantities are levels of the gene of interest in relation to quantities of housekeeping gene TOP1). (**D**) All three estrogen receptors (ERα, ERβ, and GPR30) were also present in syncytialised BeWo cells compared to nonsyncytialised BeWo cells. (**Insert in D**). Immunostaining of syncytialised BeWo cells for estrogen receptors; merged images. Green: receptor; blue: DAPI nuclear stain. ERα and ERβ show a more nuclear staining pattern, whereas GPR30 staining is more focused around the cell membrane.

BeWo cells were treated with 8-bromo-cAMP (8-Br-cAMP) for 72 h to fuse and form syncytia. E-cadherin, a cell membrane-bound protein which mediates cell-to-cell interaction, was visualized through immunofluorescence as a marker of cell membrane borders (marker of cell fusion). As trophoblasts fuse to become syncytiotrophoblasts, giant cells containing multiple nuclei and one surrounding cell membrane develop, whilst E-cadherin staining around individual cells disappears [45]. Here, we have shown that BeWo cells lose E-cadherin after treatment with 8-Br-cAMP when compared to untreated cells. There is a loss of cell membrane and a certain degree of cell fusion to form large, amorphous, multinucleated syncytia which represent the more endocrine active component of the placenta (Figure 5A).

As the syncytiotrophoblast exhibits considerable hormone-secreting properties, we measured the levels of two placental hormones, β-human chorionic gonadotrophin (β-hCG) and E2, in conditioned media of nonsyncytialised and syncytialised BeWo cells cultured for 24 h. Both hormones were significantly increased (β-hCG, *p* = 0.0108; E2, *p* = 0.004) following syncytialisation of BeWo cells with 8-Br-cAMP treatment (Figure 5B). Moreover, Syncytin-2 (a marker of syncytialisation) showed relative upregulation (more than 2-fold) in 8-Br-cAMP-treated BeWo cells compared to untreated BeWo cells (Figure 5C). All three estrogen receptors (ERα, ERβ, and GPR30) were also detected in syncytialised BeWo cells at mRNA (Figure 5D) and protein level (Figure 5E).

### 3.5. Gene Microarray Analyses Assessing the in Vitro Effects of BPA in Syncytialised BeWo Cells

Syncytialised BeWo cells were analysed separately in order to assess significantly differentially regulated genes and their functions, as well as involvement in cell signalling pathways. Overall, in syncytialised BeWo cells treated with 3 nM BPA, 309 genes were differentially regulated, whilst in those treated with 10 nM BPA, 158 genes were differentially regulated. Only one gene was commonly shared between the two BPA treatments, that is, Fatty-Acid-Binding Protein 5 (FABP5). The most significantly upregulated genes in syncytialised BeWo cells following 3 nM BPA treatment were: Growth Hormone Releasing Hormone (GHRH), followed by UDP-glucuronosyltransferase 2B10 (UGT2B10), Carbonic anhydrase-related protein 11 (CA11), Natural resistance-associated macrophage protein 1 (SLC11A1), Rab proteins geranylgeranyltransferase component A1 (CHM), OTU domain-containing protein 7A (OTUD7A), Envoplakin-like protein (EVPLL), Envoplakin-like protein (SLFNL1), Excitatory amino acid transporter 5 (SLC1A7), and Sulfotransferase 1C4 (SULT1C4).

When syncytialised BeWo cells were treated with 10 nM BPA, the most significantly upregulated genes were: Sodium-dependent phosphate transporter 2 (SLC20A2), Probable tubulin polyglutamylase (TTLL9), Arginyl-tRNA-protein transferase 1 (ATE1), Adhesion G protein-coupled receptor A2 (GPR124), Golgi-associated plant pathogenesis-related protein 1 (GLIPR2), Tumour necrosis factor receptor superfamily member 6 (FAS), Multiple inositol polyphosphate phosphatase 1 (MINPP1), Protein transport protein Sec61 subunit gamma (SEC61G), Sprouty-related EVH1 domain-containing protein 1 (SPRED1), and Carcinoembryonic antigen-related cell adhesion molecule 3 (CEACAM3). Furthermore, we have validated the SIM2 gene, at protein level, using immunofluorescence due to its role in placental physiology and/or development. Following 24 h of 10 nM BPA treatment in syncytialised BeWo cells, there was a marked increase in the expression of the SIM2 encoded protein in accordance with the microarray observations (data not shown). 

Enrichment analyses using Enrichr (using the WikiPathways 2019 database) revealed that the biological pathways most associated with differentially expressed genes in 3 nM and 10 nM BPA-treated BeWo cells are NRF2 pathway (Table 4) and mir-124 predicted interactions with cell cycle and differentiation, respectively (Table 5).

**Figure 6:**
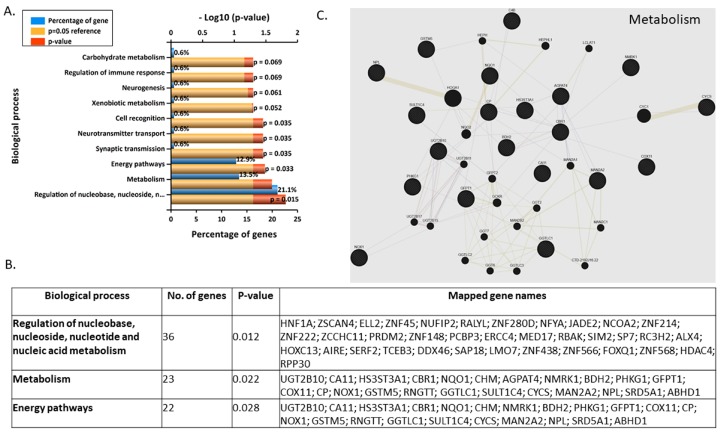
(**A**) Top 10 enriched (Funrich) biological processes for 3 nM BPA-treated syncytialised BeWos. (**B**) Table of genes involved in most significant biological processes. (**C**) Network annotation of genes involved in metabolism (Genemania; Supplementary Table S4).

Most enriched biological processes were regulation of nucleobase, nucleoside, nucleotide and nucleic acid metabolism, metabolism, and energy pathways for 3 nM BPA-treated syncytialised BeWo cells (Figure 6A,B). The 23 genes that are involved in metabolism are: UGT2B10, CA11, HS3ST3A1, CBR1, NQO1, CHM, AGPAT4, NMRK1, BDH2, PHKG1, GFPT1, COX11, CP, NOX1, GSTM5, RNGTT, GGTLC1, SULT1C4, CYCS, MAN2A2, NPL, SRD5A1, and ABHD1. These genes appear to create an extensive and diverse network (Figure 6C, Supplementary Table S3).

**Figure 7:**
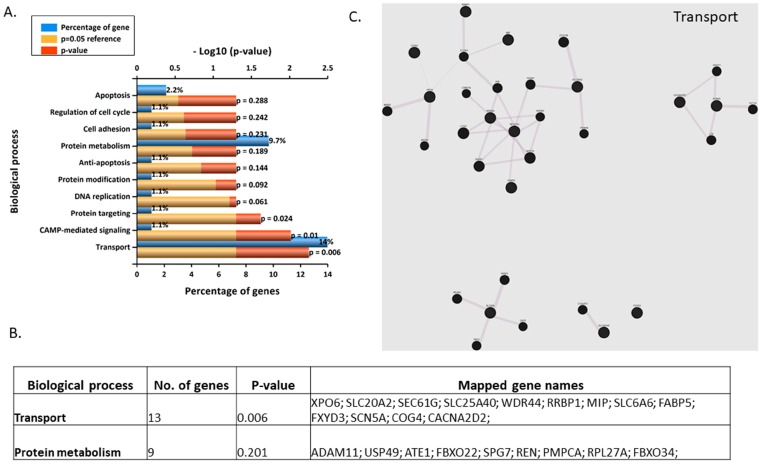
(**A**) Top 10 enriched (Funrich) biological processes for 10 nM BPA-treated syncytialised BeWos. (**B**) Table of genes involved in transport and protein metabolism.: exportin 6 (XPO6), solute carrier family 20 member 2 (SLC20A2), Sec61 translocon gamma subunit (SEC61G), solute carrier family 25 member 40 (SLC25A40), WD repeat domain 44 (WDR44), ribosome binding protein 1 (RRBP1), major intrinsic protein (MIP), solute carrier family 6 member 6 (SLC6A6), fatty acid binding protein 5 (FABP5), FXYD domain containing ion transport regulator 3 (FXYD3), sodium voltage-gated channel alpha subunit 5 (SCN5A), component of oligomeric golgi complex 4 (COG4), calcium voltage-gated channel auxiliary subunit alpha2delta 2 (CACNA2D2), ADAM metallopeptidase domain 11 (ADAM11), ubiquitin specific peptidase 49 (USP49), arginyltransferase 1 (ATE1), F-box protein 22 (FBXO22), SPG7, paraplegin matrix AAA peptidase subunit (SPG7), renin (REN), peptidase, mitochondrial processing alpha subunit (PMPCA), ribosomal protein L27a (RPL27A), F-box protein 22 (FBXO22). (**C**) Network annotation of genes involved in metabolism (Genemania).

In the case of syncytialised BeWos treated with 10 nM BPA, the most enriched biological processes were transport and cAMP-mediated signalling. However, only transport involved multiple genes (Figure 7 A,B), which were: XPO6, SLC20A2, SEC61G, SLC25A40, WDR44, RRBP1, MIP, SLC6A6, FABP5, FXYD3, SCN5A, COG4, CACNA2D2 that appear to form four distinct networks (Figure 7C, Supplementary Table S5).

## 4. Discussion

In this study, we investigated the effect of BPA using human placental cells, an in vitro model, in terms of impact on cell number and activation of key kinases in a trophoblast model in vitro. We also expanded on these observations by using whole-genome microarray analyses to study the effects of various concentrations of BPA in nonsyncytialised and syncytialised BeWo cells. 

In our studies, BPA (3 nM) significantly increased BeWo cell number, which is in line with both in vivo and in vitro studies showing that low levels of BPA increased cell proliferation in mouse pancreatic β-cells, rat dorsolateral prostate cells, rat bile duct cells, mouse spermatogonial cells, and OVCAR3 cells via different mechanisms and in a concentration-dependent manner [46,47,48,49,50]. For example, proliferation of ovarian cancer cell line OVCA3 was significantly increased when treated with 10^−9^ M but not 10^−7^ M BPA [51]. Proliferation of rat prostate epithelial cells was also significantly increased after treatment with 0.1 and 1 nM BPA, as opposed to showing decreased proliferation at 10–1000 nM of BPA [47]. The biphasic effect of BPA has also been documented in rodent models in vivo, as well as in the BeWo cell line despite using supra-physiological concentrations [43,48,52,53]. Furthermore, our data corroborate a study showing that BeWo cells treated with BPA during stress-induced conditions led to a consistent and significant increase in cell viability and a reduction in apoptosis [50]. In another study in the same in vitro model, BPA induced cell proliferation at 1 µM and decreased the proliferation rate of BeWos at 1000 µM [51]. In a more recent study, however, treatment of BeWo cells with BPA for 72 h did not affect either the proliferation or the metabolic activity [54]. However, none of these studies have provided further evidence of how BPA exerts its effects in this model. Previous studies have indicated that BPA can activate both nuclear (ERα and ERβ) and membrane (GPR30) ERs [8,10,55,56]. In this case, the proliferative effects of BPA are attributed to the activation of GPR30 rather than ERα and ERβ, since G15 (a GPR30 antagonist) but not ICI 182,780 (a pure ER antagonist) inhibited cell proliferation. These data are in direct agreement with a study in testicular seminoma cells, where a very similar effect was observed [57], where BPA acted in a nongenomic manner. Moreover, a number of studies have also reported that BPA can induce signalling cascades via GPR30 [58,59]. 

BPA can affect the phosphorylation status of numerous kinases, an effect that appears to be organ- or cell-specific. For example, both phospho-AKT and phospho-ERK1/2 have been induced by BPA in rat mammary glands [34]. On the other hand, phospho-AKT has been shown to be downregulated after BPA treatment in rat sertoli cells [51] and rat hippocampi [60]. In this study, we demonstrated that BPA can induce phosphorylation of p38 and AKT, but not ERK1/2 in BeWo cells. There was a notable decrease in cell number when both AKT and MAPK inhibitors were used, in cotreatment with BPA. In the case of the PI3K inhibitor LY294002 (LY), it is difficult to interpret the data since treatment of BeWo cells alone reduced cell number as well. However, it is worth mentioning that a similar treatment with LY resulted in increased cell fusion of BeWo cells [61]. Events that drive cell fusion will undoubtedly slow down the rate of cell growth as we have previously documented when we treated BeWo cells with forskolin [62]. Interestingly, in the same study by Vatish et al., wortmannin—a different PI3K inhibitor—did not alter the fusigenic capacity of BeWo cells, suggesting that LY might affect different pathways as well. Future studies should use a wider repertoire of intracellular signalling inhibitors to dissect these responses further.

Nonbiased microarray analyses of nonsyncytialised BeWo cells revealed some interesting targets. It should be noted that these data are novel, since, to the best of our knowledge, no other study has shown a comprehensive map of gene changes at placental level in vitro. When we compared our findings to published transcriptomic research, there was no overlap of BPA-regulated genes in endometrial or ovarian cells, suggesting that the changes observed in this study are cell-specific [63,64]. One of the most significantly upregulated genes in both 3 nM and 10 nM nonsyncytialised BeWo cells was caveolin-1 (CAV1). CAV1 is a protein that is found in caveolae, which are 50–100 nm wide invaginations of the cell lipid bilayer. The function of CAV1 in the placenta has not been fully elucidated, but it has been implicated in the transport of lipids, glucose homeostasis control, regulation of cell signalling, and membrane trafficking [65,66,67,68,69,70]. CAV1 is also involved in the palmitoylation of ERα, securing it to caveolae/lipid rafts on the cell membrane [71,72,73,74]. During pregnancy, CAV1 has been associated with glucose and fatty acid transport in the placenta by inducing AMPK and reducing the GLUT1 signalling pathway and reversing macrosomia due to gestational diabetes (GD) [74]. Furthermore, CAV1 has been implicated in the mechanism of oedema in preeclampsia (PE) following hypoxia of trophoblasts through the HMGB1/TLR4/CAV-1 pathway [75].

Another one of the most significantly upregulated genes, but not in the top 10, after treatment with 3 nM BPA was placenta-specific 1 (PLAC1). PLAC1 has been implicated in placentomegaly in mice [76], a condition which has implications in various disorders such as placental mesenchymal dysplasia [77]. Since PLAC1 upregulation induces phospho-AKT [78,79,80], this might be a potential mechanism at placental level that can drive upregulation of phospho-AKT and, thus, potentially promote cell proliferation. Arguably this is a limitation in the study (i.e., to provide definitive proof of the involvement of this gene in AKT phosphorylation in this cellular model). Future studies overexpressing or silencing PLAC1 followed by assessment of the phosphorylation status of key kinases should provide a novel insight. 

Another upregulated gene after BPA treatment was leptin. It is well documented that placental leptin is modulated by numerous hormones and cytokines [81,82]. Leptin is involved in the implantation process of the embryo by increasing trophoblast matrix metalloproteinase expression, allowing for better cell invasion [83,84,85]. Leptin has also been implicated in many pathologies, and there is a correlation of maternal plasma leptin levels and the development of GD [86,87,88,89,90]. For example, leptin levels were found raised in the GD group of pregnant women, even when adjusting for confounders [86]. Higher serum leptin levels in pregnant women with PE have also been documented [91,92], and there is an association between higher leptin expression and intrauterine growth restriction (IUGR) [93,94]. To date, a few studies have shown a relationship between BPA and leptin. For example, BPA associated positively with adiponectin and leptin, but negatively with ghrelin, following adjustments for sex, height, fat mass, lean mass, smoking, alcohol consumption, physical activity, energy intake, and educational levels in 890 elderly men and women [95]. Moreover, in 3T3-L1 adipocytes differentiated in the presence of physiological concentrations of BPA, there was an increase in the expression of leptin, IL-6, and interferon-γ [96].

Placenta-specific protein 8 (PLAC8) was also a significantly upregulated gene in both 3 nM and 10 nM BPA-treated nonsyncytialised BeWo. PLAC8 is expressed on the feto-maternal interface, where it plays a role in promoting trophoblast invasion and migration and is significantly upregulated under hypoxic conditions and in PE placentae [97]. PLAC8 is implicated in diseases such as obesity, type 2 diabetes, and GD [98]. PLAC8 was found to be highly expressed in neonatal cells exposed to GD and expression of PLAC8 was correlated with maternal hyperglycemia [99]. PLAC8 also plays a role in adipogenesis, brown fat differentiation, and body weight control by controlling C/EBPβ expression [100,101]. In addition, overexpression of PLAC8 leads to increased growth, resistance to apoptosis, and higher levels of phosphorylated Akt1 in fibroblasts [102]. 

The targets in both 3 nM and 10 nM BPA-treated syncytialised BeWo cells were different, indicative of the syncytialisation process and the changes in terms of the endocrine/signalling milieu or the activation of the cAMP/PKA/CEB pathway. GHRH was the most upregulated gene in 3 nM treated cells. Although the expression of GHRH in the human placenta is documented, its exact role is still poorly investigated [103], with its levels being elevated in the third trimester. In a more recent study in another placental in vitro model (JEG-3 cells), inhibition of GHRH-R by a GHRH antagonist reduced cell viability and induced apoptosis through inactivation of Akt [104]. It will be interesting to repeat the experiment in syncytialised BeWo cells in order to gain a better understanding of whether this hormone alters Akt phosphorylation.

Slc11a1 (solute carrier family 11 member 1), the gene that encodes for the Natural resistance-associated macrophage protein 1, is upregulated in BPA-treated syncytialised BeWo cells and plays a role in host innate immunity. This represents a divalent cation transporter which is expressed primarily by macrophages and neutrophils and is essential for controlling infections by intracellular pathogens, with previous studies showing its expression in the syncytiotrophoblast of the human placenta at multiple gestational ages [105]. Moreover, the Single-Minded 2 (SIM2) gene was significantly upregulated. This, as a basic helix–loop–helix (bHLH) protein, belongs to a group of transcription factors that regulates several downstream genes involved in developmental and neurological pathways [106], playing a role in proliferation and in embryo development [107].

Regarding the role of FABP5 (Fatty-Acid-Binding Protein 5), the only gene that was common between the two treatments, very little is known regarding its role at placental level. A single study demonstrates that FABP5 mRNA expression was reduced in placental macrovascular endothelial cells of obese versus lean women, but not in trophoblasts [108]. This finding underpins once again the novelty of our microarray data. Future studies should involve silencing FABP5 to dissect further its role in placentation. 

Taken together, these findings highlight the capacity of BPA to affect the BeWo cell genome. The most significant changes were seen in cells that appeared to be most susceptible to BPA treatment, that is, 3 nM treated nonsyncytialised BeWo cells. These changes imply a role of BPA in influencing the metabolism, as well as the number of placental cells, factors that could significantly affect fetal and placental development and determine the outcome of the pregnancy itself. Insulin signalling is a pathway which has been demonstrated to play a major role during pregnancy and in diseases, such as GD, which can have severe effects on the fetus and pregnancy, and long-term effects on both mother and child (e.g., fetal macrosomia, maternal PE, neonatal hyperglycemia, respiratory distress syndrome, and development of type 2 diabetes of the mother after pregnancy, as well as increased risk of obesity and abnormal glucose metabolism of the offspring later in life) [109,110,111].

In conclusion, we provide novel evidence that BPA can potentially affect mechanisms implicated in a number of different processes in vitro, even in low nanomolar concentrations. These in vitro findings warrant further investigation in order to elucidate the exact impact of this EDC in fetal programming.

## Figures and Tables

**Table 1:** List of primers used for qRT-PCR.

Gene	Sequence
CAV1	F: 5′ACCCACTCTTTGAAGCTGTTG3′
	R: 5′GAACTTGAAATTGGCACCAGG3′
Leptin	F: 5′CCTGACTGGTGCTATAGGCTGGA3′
	R: 5′GTGAGTGCGGTTTGACCACTG3′
hPLAC8	F: 5′GGGTGTCAAGTTGCAGCTGAT3′
	R: 5′TAGATCCAGGGATGCCATATCG3′
Syncytin 2	F: 5′AGCAGCCGTAGTCCTTCAAA3′
	R: 5′AGGGGAAGAACCCAAGAGAA3′
ERα	F: 5′GCCCTCCCTCCCTGAAC3′
	R: 5′TCAACTACCATTTACCCTCATC3′
ERβ	F: 5′TCCTCCCAGCAGCAATCC3′
	R: 5′CCAGCAGCAGGTCATACAC3′
GPR30	F: 5′GTTCCTCTCGTGCCTCTAC3′
	R: 5′ACCGCCAGGTTGATGAAG3′
TOP1	F: 5′CCTTCCCTCTCTCCCATTTC3′
	R: 5′AGCCACGACTGCTTCAAGTT3′

CAV1: Caveolin 1, hPLAC8: Placenta-specific gene 8 protein, ERα: Estrogen receptor-alpha, ERβ: Estrogen receptor-beta, GPR30: membrane-bound estrogen receptor, TOP1: DNA topoisomerase I.2.4. Microarray.

**Table 2:** Top 10 pathways associated with differentially expressed genes after 3 nM bisphenol A (BPA) treatment of BeWo cells using the WikiPathways 2019 Human database (Enrichr). The most significantly regulated pathway is leptin/insulin overlap.

Index	Biological Pathway	*p* Value	Input Genes
1	Leptin/insulin overlap	0.0016	suppressor of cytokine signaling 3 (SOCS3), suppressor of cytokine signaling 1 (SOCS1), leptin (LEP), insulin receptor substrate 2 (IRS2)
2	Differentiation of white and brown adipocyte	0.0098	PLAC8-like 1 (PLAC8), LEP, SMAD family member 9 (SMAD9), zinc finger protein 423 (ZNF423), PPARG coactivator 1 beta (PPARGC1B)
3	Kit receptor signalling pathway	0.0136	ribosomal protein S6 kinase, 90 kDa, polypeptide 3 (RPS6KA3), (mitogen-activated protein kinase 8) MAPK8, SOCS1, ribosomal protein S6 kinase B1 (RPS6KB1), SHC (Src homology 2 domain containing) transforming protein 1 (SHC1), inositol polyphosphate-5-phosphatase D (INPP5D), FYN proto-oncogene, Src family tyrosine kinase (FYN), microtubule-associated protein tau (MAPT)
4	Integrin-mediated cell adhesion	0.0205	vasodilator-stimulated phosphoprotein (VASP), G protein-coupled receptor kinase interacting ArfGAP 2 (GIT2), (SHC1), Rho-associated, coiled-coil containing protein kinase 2 (ROCK2), CAV1, integrin, alpha 1 (ITGA1), integrin, alpha X (complement component 3 receptor 4 subunit) (ITGAX), Rap guanine nucleotide exchange factor (GEF) 1 (RAPGEF1), p21 protein (Cdc42/Rac)-activated kinase 6 (PAK6), integrin, alpha 6 (ITGA6), FYN
5	Prolactin signalling pathway	0.0211	SOCS3, MAPK8, SOCS1, RPS6KB1, SHC1, erb-b2 receptor tyrosine kinase 2 (ERBB2), IRS2, FYN
6	Leptin signalling pathway	0.0211	SOCS3, MAPK8, RPS6KB1, SOCS1, SHC1, ROCK2, LEP, ERBB2, BCL2-associated X protein (BAX), FYN
7	IL-2 signalling pathway	0.0244	SOCS3, RPS6KB1, SHC1, FYN, N-myc (and STAT) interactor (NMI), MAPT
8	Angiopoietin-like protein 8 regulatory pathway	0.0265	SHC1, solute carrier family 2 (facilitated glucose transporter), member 1 (SLC2A1), protein kinase, AMP-activated, gamma 1 non-catalytic subunit (PRKAG1), phosphoinositide-3-kinase, regulatory subunit 3 (gamma) (PIK3R3), IRS2, RPS6KA3, MAPK8, sestrin 3 (SESN3), RPS6KB1, RAPGEF1, mitogen-activated protein kinase kinase kinase 6 (MAP3K6), son of sevenless homolog 2 (SOS2), mitogen-activated protein kinase kinase kinase 5 (MAP3K5)
9	Insulin signalling	0.0268	syntaxin binding protein 4 (STXBP4), SHC1, SLC2A1, PIK3R3, RPS6KA3, SOCS3, MAPK8, SOCS1, RPS6KB1, RAPGEF1, tribbles pseudokinase 3 (TRIB3), MAP3K6, SOS2, MAP3K5
10	Focal adhesion	0.0403	vasodilator-stimulated phosphoprotein (VASP), von Willebrand factor (VWF), SHC1, ROCK2, laminin, beta 2 (laminin S) (LAMB2), CAV1, ITGA1, PIK3R3, Rho GTPase activating protein 5 (ARHGAP5), myosin light chain kinase (MYLK), MAPK8, ERBB2, RAPGEF1, p21 protein (Cdc42/Rac)-activated kinase 6 (PAK6), filamin B, beta (FLNB), ITGA6, FYN

**Table 3:** Top 10 significant biological pathways associated with differentially expressed genes after 10 nM bisphenol A (BPA) treatment of BeWo cells using the WikiPathways 2019 Human database (Enrichr). The most significantly regulated pathway is differentiation of white and brown adipocyte.

Index	Term	*p* Value	Input Genes
1	Differentiation of white and brown adipocyte	0.002	PLAC8, SMAD family member 1 (SMAD1), LEP, PPARGC1B
2	Methylation pathways	0.015	nicotinamide N-methyltransferase (NNMT), thiopurine S-methyltransferase (TPMT)
3	Mechanoregulation and pathology of YAP/TAZ via Hippo and non-Hippo mechanisms	0.019	integrin, beta 4 (ITGB4), integrin, beta 3 (ITGB3), macrophage stimulating 1 (MST1), actin, gamma 2 (ACTG2)
4	Photodynamic therapy-induced unfolded protein response	0.021	DNA-damage-inducible transcript 3 (DDIT3), TRIB3, activating transcription factor 3 (ATF3)
5	Alanine and aspartate metabolism	0.027	alanine-glyoxylate aminotransferase (AGXT), argininosuccinate synthase 1 (ASS1)
6	Mitochondrial gene expression WP391	0.064	GA binding protein transcription factor, beta subunit 1(GABPB1), PPARGC1B
7	Hypertrophy model	0.070	ATF3, heparin-binding EGF-like growth factor (HBEGF)
8	Complement activation	0.083	Complement C 3 (C3), C15
9	Exercise-induced circadian regulation	0.087	DAZ associated protein 2 (DAZAP2), cryptochrome circadian clock 2 (CRY2)
(10	NRF2 pathway	0.101	ATP-binding cassette, sub-family C (CFTR/MRP), member 3 (ABCC3), early growth response 1 (EGR1), solute carrier family 6 member 9 (SLC6A9), glutathione S-transferase alpha 4 (GSTA4), solute carrier family 39 member 7 SLC39A7, HBEGF

**Table 4:** Top 10 biological pathways associated with differentially expressed genes after 3 nM bisphenol A (BPA) treatment of syncytialised BeWo cells using the WikiPathways 2019 database (Enrichr).

Index	Name of Biological Pathway	*p*-Value	Input Genes
1	nuclear factor erythroid 2-related factor 2 (NRF2) pathway	0.00464	ATP-binding cassette, sub-family C (CFTR/MRP), member 3 (ABCC3); gamma-glutamyltransferase light chain 1 (GGTLC1); carbonyl reductase 1 (CBR1); NAD(P)H dehydrogenase, quinone 1 (NQO1); solute carrier family 6 member 15 (SLC6A15); solute carrier family 2 member 5 (SLC2A5); glutathione S-transferase mu 5 (GSTM5)
2	Role of Osx and miRNAs in tooth development	0.01831	HNF1 homeobox A (HNF1A); Sp7 transcription factor (SP7)
3	Oxidative damage	0.01849	B-cell CLL/lymphoma 2 (BCL2); cytochrome c, somatic (CYCS); tumor necrosis factor receptor superfamily, member 1B (TNFRSF1B)
4	Nanomaterial-induced apoptosis	0.03164	BCL2; CYCS
5	Nuclear receptors meta-pathway	0.03703	ABCC3; gamma-glutamyltransferase light chain 1 (GGTLC1); carbonyl reductase 1 (CBR1); nuclear receptor coactivator 2 (NCOA2); NQO1; SC6A15; DnaJ (Hsp40) homolog, subfamily C, member 15 (DNAJC15); SLC2A5; GSTM5
6	Apoptosis modulation and signalling	0.03943	BCL2; CYCS; Bcl2 modifying factor (BMF); tumor necrosis factor receptor superfamily, member 1B (TNFRSF1B)
7	Photodynamic therapy-induced NFE2L2 (NRF2) survival signalling	0.04431	ABCC3; NQO1
8	Gastric cancer network 2	0.07007	collagen, type IX, alpha 1(COL9A1); family with sequence similarity 91, member A1 (FAM91A1)
9	Constitutive androstane receptor pathway	0.07406	ABCC3; NCOA2
10	Oxidative stress	0.07814	NQO1; NADPH oxidase 1 (NOX1)

**Table 5:** Top 10 biological pathways most significantly associated with differentially expressed genes after 10 nM bisphenol A (BPA) treatment of syncytialised BeWo cells using the WikiPathways 2019 database (Enrichr).

Index	Name of Biological Pathway	*p*-Value	Input Genes
1	mir-124 predicted interactions with cell cycle and differentiation	0.0395	STE20-related kinase adaptor beta (STRADB)
2	LncRNA-mediated mechanisms of therapeutic resistance	0.0395	hypoxia inducible factor 1, alpha subunit (HIF1A)
3	MicroRNA for targeting cancer growth and vascularization	0.0460	HIF1A
4	HIF1A and Peroxisome Proliferator Activated Receptor Gamma (PPARG) regulation of glycolysis	0.0524	HIF1A
5	TGIF disruption of SHH signalling (Hedgehog signaling pathway)	0.0587	TGFB-induced factor homeobox 1 (TGIF1)
6	Notch signalling pathway	0.0631	melanoma antigen family A,1 (MAGEA1); HIF1A
7	Steroid biosynthesis	0.0650	hydroxysteroid (17-beta) dehydrogenase 4 (HSD17B4)
8	Ectoderm differentiation	0.0657	frizzled class receptor 5 (FZD5); ribosome binding protein 1 (RRBP1); WD repeat domain 44 (WDR44)
9	G1 to S cell cycle control	0.0686	cyclin G2 (CCNG2); polymerase (DNA directed), epsilon, catalytic subunit (POLE)
10	NAD metabolism, sirtuins, and aging	0.0713	HIF1A

## Supplementary Materials

The following are available online at https://www.mdpi.com/2077-0383/9/2/405/s1, Table S1: Interactions of genes involved in metabolism in 3 nM BPA-treated non-ST-BeWos, Table S2: Interactions of genes involved in metabolism in 10 nM BPA-treated non-ST-BeWos, Table S3: Interactions of genes involved in metabolism in 3 nM BPA-treated ST-BeWos, Table S4: Acronyms of genes involved in regulation of nucleic acid metabolism, metabolism and energy pathways, Table S5: Interactions of genes involved in metabolism in 10 nM BPA treated ST-BeWos.
