# Supplementary material for: Involvement of the Endocrine-Disrupting Chemical Bisphenol A (BPA) in Human Placentation
Source: J Clin Med. 2020 Feb 3;9(2):405. doi: 10.3390/jcm9020405 (PMC7074564; doi:10.3390/jcm9020405)
Supplement: Supplementary file 1 [file jcm-09-00405-s001.pdf]

Supplementary Table S1:

Interactions of genes involved in metabolism in 3nM BPA treated non-ST-BeWos.

Gene 1	Gene 2	Interaction	Network
PTBP3	CDCA4	Co-expression	Ramaswamy-Golub-2001
NT5C1A	CASZ1	Co-expression	Mallon-McKay-2013
FAM118A	CASZ1	Co-expression	Dobbin-Giordano-2005
SERTAD2	SERTAD3	Co-expression	Bahr-Bowler-2013
SERTAD3	SERTAD1	Co-expression	Noble-Diehl-2008
SERTAD2	SERTAD1	Co-expression	Noble-Diehl-2008
FAM118A	CASZ1	Co-expression	Noble-Diehl-2008
SEMA3G	HCFC1R1	Co-expression	Noble-Diehl-2008
SERTAD3	SERTAD1	Co-expression	Roth-Zlotnik-2006
IRX4	CASZ1	Co-expression	Roth-Zlotnik-2006
OVOL2	CASZ1	Co-expression	Roth-Zlotnik-2006
PTBP3	CDCA4	Co-expression	Roth-Zlotnik-2006
PTBP3	SERTAD2	Co-expression	Roth-Zlotnik-2006
LY6G5C	DOK5	Co-expression	Smirnov-Cheung-2009
LY6G5C	GLP2R	Co-expression	Smirnov-Cheung-2009
SERTAD2	SERTAD3	Co-expression	Wang-Cheung-2015
GLP2R	CASZ1	Co-expression	Wang-Cheung-2015
IRX4	CASZ1	Co-localization	Johnson-Shoemaker-2003
GLP2R	CASZ1	Co-localization	Johnson-Shoemaker-2003
OVOL2	CASZ1	Co-localization	Johnson-Shoemaker-2003
LY6G5C	CASZ1	Co-localization	Johnson-Shoemaker-2003
LY6G5C	OVOL2	Co-localization	Johnson-Shoemaker-2003
EEF2K	CASZ1	Co-localization	Johnson-Shoemaker-2003
HCFC1R1	CASZ1	Co-localization	Johnson-Shoemaker-2003
SEMA3G	CASZ1	Co-localization	Johnson-Shoemaker-2003
PPP2R2A	CDCA4	Physical Interactions	Hein-Mann-2015
TEAD2	CASZ1	Physical Interactions	Li-Chen-2015
SUPT7L	CDCA4	Physical Interactions	BIOGRID-SMALL-SCALE-STUDIES
SUPT7L	SERTAD1	Physical Interactions	BIOGRID-SMALL-SCALE-STUDIES
SUPT7L	SERTAD2	Physical Interactions	BIOGRID-SMALL-SCALE-STUDIES
PPP2R2A	SERTAD4	Physical Interactions	Huttlin-Gygi-2015
PPP2R2C	CDCA4	Physical Interactions	Huttlin-Gygi-2015
PPP2R2C	SERTAD4	Physical Interactions	Huttlin-Gygi-2015
SEPHS1	CDCA4	Physical Interactions	IREF-INTACT
DOK5	CDCA4	Physical Interactions	Grossmann-Stelzl-2015
SEPHS1	CDCA4	Physical Interactions	Yu-Vidal-2011
SERTAD4	CDCA4	Shared protein domains	INTERPRO
SERTAD1	CDCA4	Shared protein domains	INTERPRO
SERTAD1	SERTAD4	Shared protein domains	INTERPRO
SERTAD3	CDCA4	Shared protein domains	INTERPRO
SERTAD3	SERTAD4	Shared protein domains	INTERPRO
SERTAD3	SERTAD1	Shared protein domains	INTERPRO
SERTAD2	CDCA4	Shared protein domains	INTERPRO
SERTAD2	SERTAD4	Shared protein domains	INTERPRO
SERTAD2	SERTAD1	Shared protein domains	INTERPRO

SERTAD2	SERTAD3	Shared protein domains	INTERPRO
PPP2R2C	PPP2R2A	Shared protein domains	INTERPRO
OVOL2	CASZ1	Shared protein domains	INTERPRO
SERTAD4	CDCA4	Shared protein domains	PFAM
SERTAD1	CDCA4	Shared protein domains	PFAM
SERTAD1	SERTAD4	Shared protein domains	PFAM
SERTAD3	CDCA4	Shared protein domains	PFAM
SERTAD3	SERTAD4	Shared protein domains	PFAM
SERTAD3	SERTAD1	Shared protein domains	PFAM
SERTAD2	CDCA4	Shared protein domains	PFAM
SERTAD2	SERTAD4	Shared protein domains	PFAM
SERTAD2	SERTAD1	Shared protein domains	PFAM
SERTAD2	SERTAD3	Shared protein domains	PFAM

Supplementary Table S2:

Interactions of genes involved in metabolism in 10nM BPA treated non-ST-BeWos

Gene 1	Gene 2	Interaction	Network
PMP22	CD151	Co-expression	Ramaswamy-Golub-2001
DST	PCDH1	Co-expression	Ramaswamy-Golub-2001
MST1R	COL17A1	Co-expression	Ramaswamy-Golub-2001
LAMC2	MMP7	Co-expression	Ramaswamy-Golub-2001
CD151	PLEC	Co-expression	Wang-Maris-2006
DST	PMP22	Co-expression	Wang-Maris-2006
LAMC2	COL17A1	Co-expression	Wang-Maris-2006
LAMC2	MMP7	Co-expression	Wang-Maris-2006
MST1R	ITGB4	Co-expression	Mallon-McKay-2013
PLEC	ITGB4	Co-expression	Bild-Nevins-2006 B
CD151	PCDH1	Co-expression	Bild-Nevins-2006 B
DST	COL17A1	Co-expression	Bild-Nevins-2006 B
YES1	ITGA6	Co-expression	Bild-Nevins-2006 B
ERBB2	ITGB4	Co-expression	Bild-Nevins-2006 B
LAMA3	COL17A1	Co-expression	Bild-Nevins-2006 B
LAMA3	DST	Co-expression	Bild-Nevins-2006 B
LAMC2	COL17A1	Co-expression	Bild-Nevins-2006 B
LAMC2	CLCA1	Co-expression	Bild-Nevins-2006 B
MET	CLCA1	Co-expression	Burington-Shaughnessy-2008
MST1R	PCDH1	Co-expression	Burington-Shaughnessy-2008
CLCA2	COL17A1	Co-expression	Dobbin-Giordano-2005
CLCA2	ITGA6	Co-expression	Dobbin-Giordano-2005
MET	CD151	Co-expression	Dobbin-Giordano-2005
LAMA3	COL17A1	Co-expression	Dobbin-Giordano-2005
LAMA3	CLCA2	Co-expression	Dobbin-Giordano-2005
LAMC2	COL17A1	Co-expression	Dobbin-Giordano-2005
LAMC2	CLCA2	Co-expression	Dobbin-Giordano-2005
LAMC2	LAMA3	Co-expression	Dobbin-Giordano-2005
YES1	ITGB4	Co-expression	Alizadeh-Staudt-2000
ERBB2	ITGB4	Co-expression	Alizadeh-Staudt-2000

VIM	CD151	Co-expression	Alizadeh-Staudt-2000
LAMA3	COL17A1	Co-expression	Innocenti-Brown-2011
MST1R	ITGB4	Co-expression	Innocenti-Brown-2011
LAMC2	ITGB4	Co-expression	Innocenti-Brown-2011
LAMC2	MMP7	Co-expression	Innocenti-Brown-2011
MST1R	ITGB4	Co-expression	Noble-Diehl-2008
MST1R	DST	Co-expression	Noble-Diehl-2008
MST1R	LAMA3	Co-expression	Noble-Diehl-2008
LAMC2	LAMA3	Co-expression	Noble-Diehl-2008
LAMC2	MST1R	Co-expression	Noble-Diehl-2008
CLCA2	COL17A1	Co-expression	Roth-Zlotnik-2006
LAMC2	MMP7	Co-expression	Roth-Zlotnik-2006
ITGA6	CD151	Co-expression	Boldrick-Relman-2002
ERBB2	COL17A1	Co-expression	Boldrick-Relman-2002
COL17A1	ITGB4	Co-expression	Perou-Botstein-2000
ITGA6	ITGB4	Co-expression	Perou-Botstein-2000
ITGA6	COL17A1	Co-expression	Perou-Botstein-2000
DST	ITGB4	Co-expression	Perou-Botstein-2000
DST	COL17A1	Co-expression	Perou-Botstein-2000
DST	ITGA6	Co-expression	Perou-Botstein-2000
VIM	PMP22	Co-expression	Perou-Botstein-2000
LAMA3	ITGB4	Co-expression	Perou-Botstein-2000
LAMA3	COL17A1	Co-expression	Perou-Botstein-2000
LAMA3	ITGA6	Co-expression	Perou-Botstein-2000
LAMA3	DST	Co-expression	Perou-Botstein-2000
LAMA3	MET	Co-expression	Perou-Botstein-2000
MST1R	ITGB4	Co-expression	Perou-Botstein-2000
LAMC2	ITGB4	Co-expression	Perou-Botstein-2000
LAMC2	CD151	Co-expression	Perou-Botstein-2000
LAMC2	COL17A1	Co-expression	Perou-Botstein-2000
LAMC2	ITGA6	Co-expression	Perou-Botstein-2000
LAMC2	DST	Co-expression	Perou-Botstein-2000
LAMC2	LAMA3	Co-expression	Perou-Botstein-2000
LAMC2	MST1R	Co-expression	Perou-Botstein-2000
CD151	PLEC	Co-expression	Wang-Cheung-2015
COL17A1	ITGB4	Co-expression	Wang-Cheung-2015
ITGA6	COL17A1	Co-expression	Wang-Cheung-2015
ERBB2	PLEC	Co-expression	Wang-Cheung-2015
CLCA2	ITGB4	Co-expression	Wang-Cheung-2015
CLCA2	COL17A1	Co-expression	Wang-Cheung-2015
CLCA2	ITGA6	Co-expression	Wang-Cheung-2015
LAMA3	ITGB4	Co-expression	Wang-Cheung-2015
LAMA3	COL17A1	Co-expression	Wang-Cheung-2015
LAMA3	ITGA6	Co-expression	Wang-Cheung-2015
LAMA3	CLCA2	Co-expression	Wang-Cheung-2015
LAMC2	PCDH1	Co-expression	Wang-Cheung-2015
LAMC2	ITGB4	Co-expression	Wang-Cheung-2015
LAMC2	COL17A1	Co-expression	Wang-Cheung-2015
LAMC2	ITGA6	Co-expression	Wang-Cheung-2015

LAMC2	MMP7	Co-expression	Wang-Cheung-2015
LAMC2	CLCA2	Co-expression	Wang-Cheung-2015
LAMC2	LAMA3	Co-expression	Wang-Cheung-2015
CD151	PLEC	Co-expression	Chen-Brown-2002
MST1R	COL17A1	Co-expression	Chen-Brown-2002
LAMC2	ITGB4	Co-expression	Chen-Brown-2002
LAMC2	MMP7	Co-expression	Chen-Brown-2002
LAMC2	MST1R	Co-expression	Chen-Brown-2002
VIM	MMP7	Co-expression	Perou-Botstein-1999
MST1R	ITGA6	Co-expression	Perou-Botstein-1999
LAMC2	ITGA6	Co-expression	Perou-Botstein-1999
LAMC2	MST1R	Co-expression	Perou-Botstein-1999
YES1	DST	Co-expression	Wu-Garvey-2007
CLCA1	CLCA2	Co-expression	Wu-Garvey-2007
MST1R	CD151	Co-expression	Rosenwald-Staudt-2001
DST	PMP22	Co-localization	Schadt-Shoemaker-2004
LAMA3	ITGA6	Co-localization	Schadt-Shoemaker-2004
ITGA6	ITGB4	Co-localization	Johnson-Shoemaker-2003
CLCA1	ITGA6	Co-localization	Johnson-Shoemaker-2003
LAMA3	ITGB4	Co-localization	Johnson-Shoemaker-2003
MST1R	ITGA6	Co-localization	Johnson-Shoemaker-2003
LAMC2	LAMA3	Co-localization	Johnson-Shoemaker-2003
PLEC	ITGB4	Pathway	Wu-Stein-2010
EIF6	ITGB4	Pathway	Wu-Stein-2010
COL17A1	ITGB4	Pathway	Wu-Stein-2010
LAMC3	ITGB4	Pathway	Wu-Stein-2010
LAMC3	COL17A1	Pathway	Wu-Stein-2010
ITGA6	ITGB4	Pathway	Wu-Stein-2010
ITGA6	CD151	Pathway	Wu-Stein-2010
ITGA6	COL17A1	Pathway	Wu-Stein-2010
ITGA6	LAMC3	Pathway	Wu-Stein-2010
PMP22	ITGB4	Pathway	Wu-Stein-2010
PMP22	LAMC3	Pathway	Wu-Stein-2010
PMP22	ITGA6	Pathway	Wu-Stein-2010
DST	ITGB4	Pathway	Wu-Stein-2010
YES1	ITGB4	Pathway	Wu-Stein-2010
ERBB2	ITGB4	Pathway	Wu-Stein-2010
ERBB2	ITGA6	Pathway	Wu-Stein-2010
MMP7	ITGB4	Pathway	Wu-Stein-2010
VIM	ITGB4	Pathway	Wu-Stein-2010
MET	ITGB4	Pathway	Wu-Stein-2010
MET	ITGA6	Pathway	Wu-Stein-2010
LAMA3	ITGB4	Pathway	Wu-Stein-2010
LAMA3	COL17A1	Pathway	Wu-Stein-2010
LAMA3	LAMC3	Pathway	Wu-Stein-2010
LAMA3	ITGA6	Pathway	Wu-Stein-2010
LAMA3	PMP22	Pathway	Wu-Stein-2010
LAMC2	ITGB4	Pathway	Wu-Stein-2010
LAMC2	COL17A1	Pathway	Wu-Stein-2010

LAMC2	ITGA6	Pathway	Wu-Stein-2010
LAMC2	PMP22	Pathway	Wu-Stein-2010
LAMC2	LAMA3	Pathway	Wu-Stein-2010
PLEC	ITGB4	Pathway	REACTOME
CD151	ITGB4	Pathway	REACTOME
CD151	PLEC	Pathway	REACTOME
COL17A1	ITGB4	Pathway	REACTOME
COL17A1	PLEC	Pathway	REACTOME
COL17A1	CD151	Pathway	REACTOME
LAMC3	ITGB4	Pathway	REACTOME
ITGA6	PLEC	Pathway	REACTOME
ITGA6	CD151	Pathway	REACTOME
ITGA6	COL17A1	Pathway	REACTOME
ITGA6	LAMC3	Pathway	REACTOME
LAMA3	PLEC	Pathway	REACTOME
LAMA3	CD151	Pathway	REACTOME
LAMA3	COL17A1	Pathway	REACTOME
LAMC2	PLEC	Pathway	REACTOME
LAMC2	CD151	Pathway	REACTOME
LAMC2	COL17A1	Pathway	REACTOME
ITGA6	ITGB4	Pathway	NCI_NATURE
PMP22	ITGB4	Pathway	NCI_NATURE
PMP22	ITGA6	Pathway	NCI_NATURE
MET	ITGA6	Pathway	NCI_NATURE
LAMA3	ITGB4	Pathway	NCI_NATURE
LAMA3	ITGA6	Pathway	NCI_NATURE
MST1R	ITGB4	Pathway	NCI_NATURE
MST1R	ITGA6	Pathway	NCI_NATURE
LAMC2	ITGB4	Pathway	NCI_NATURE
LAMC2	ITGA6	Pathway	NCI_NATURE
LAMC2	LAMA3	Pathway	NCI_NATURE
YES1	ITGB4	Pathway	CELL_MAP
ERBB2	ITGB4	Pathway	CELL_MAP
MMP7	ITGB4	Pathway	CELL_MAP
CLCA2	ITGB4	Pathway	CELL_MAP
CLCA1	ITGB4	Pathway	CELL_MAP
VIM	ITGB4	Pathway	CELL_MAP
MET	ITGB4	Pathway	CELL_MAP
MET	YES1	Physical Interactions	Leung-Jones-2014
EIF6	HPRT1	Physical Interactions	Kristensen-Foster-2012
PLEC	ITGB4	Physical Interactions	Havugimana-Emili-2012
HPRT1	FAT3	Physical Interactions	Havugimana-Emili-2012
RNF14	PCDH1	Physical Interactions	Hein-Mann-2015
EIF6	ITGB4	Physical Interactions	BIOGRID-SMALL-SCALE-STUDIES
COL17A1	ITGB4	Physical Interactions	BIOGRID-SMALL-SCALE-STUDIES
DST	ITGB4	Physical Interactions	BIOGRID-SMALL-SCALE-STUDIES
DST	COL17A1	Physical Interactions	BIOGRID-SMALL-SCALE-STUDIES
VIM	PLEC	Physical Interactions	BIOGRID-SMALL-SCALE-STUDIES
ITGA6	ITGB4	Physical Interactions	IREF-PUBMED

PLEC	ITGB4	Physical Interactions	IREF-INTACT
ITGA6	ITGB4	Physical Interactions	IREF-INTACT
MET	YES1	Physical Interactions	IREF-INTACT
PLEC	ITGB4	Physical Interactions	IREF-HPRD
EIF6	ITGB4	Physical Interactions	IREF-HPRD
COL17A1	ITGB4	Physical Interactions	IREF-HPRD
COL17A1	PLEC	Physical Interactions	IREF-HPRD
ITGA6	ITGB4	Physical Interactions	IREF-HPRD
ITGA6	PLEC	Physical Interactions	IREF-HPRD
ITGA6	CD151	Physical Interactions	IREF-HPRD
ITGA6	COL17A1	Physical Interactions	IREF-HPRD
DST	ITGB4	Physical Interactions	IREF-HPRD
DST	COL17A1	Physical Interactions	IREF-HPRD
YES1	ITGB4	Physical Interactions	IREF-HPRD
ERBB2	ITGB4	Physical Interactions	IREF-HPRD
MMP7	CD151	Physical Interactions	IREF-HPRD
CLCA2	ITGB4	Physical Interactions	IREF-HPRD
CLCA1	ITGB4	Physical Interactions	IREF-HPRD
VIM	ITGB4	Physical Interactions	IREF-HPRD
VIM	PLEC	Physical Interactions	IREF-HPRD
MET	ITGB4	Physical Interactions	IREF-HPRD
MST1R	YES1	Physical Interactions	IREF-HPRD
CD151	ITGB4	Predicted	Wu-Stein-2010
MST1R	YES1	Predicted	Wu-Stein-2010
LAMC3	PLEC	Predicted	Stuart-Kim-2003
DST	PLEC	Shared protein domains	INTERPRO
ERBB2	YES1	Shared protein domains	INTERPRO
CLCA1	CLCA2	Shared protein domains	INTERPRO
MET	YES1	Shared protein domains	INTERPRO
MET	ERBB2	Shared protein domains	INTERPRO
LAMA3	FAT3	Shared protein domains	INTERPRO
LAMA3	LAMC3	Shared protein domains	INTERPRO
MST1R	YES1	Shared protein domains	INTERPRO
MST1R	ERBB2	Shared protein domains	INTERPRO
MST1R	MET	Shared protein domains	INTERPRO
LAMC2	LAMC3	Shared protein domains	INTERPRO
LAMC2	LAMA3	Shared protein domains	INTERPRO
DST	PLEC	Shared protein domains	PFAM
CLCA1	CLCA2	Shared protein domains	PFAM
MET	ERBB2	Shared protein domains	PFAM
LAMA3	FAT3	Shared protein domains	PFAM
LAMA3	LAMC3	Shared protein domains	PFAM
MST1R	ERBB2	Shared protein domains	PFAM
MST1R	MET	Shared protein domains	PFAM
LAMC2	LAMC3	Shared protein domains	PFAM
LAMC2	LAMA3	Shared protein domains	PFAM

Supplementary Table S3:

Interactions of genes involved in metabolism in 3nM BPA treated ST-BeWos

Gene 1	Gene 2	Interaction	Network
NQO1	GFPT1	Co-expression	Dobbin-Giordano-2005
CBR1	BDH2	Co-expression	Dobbin-Giordano-2005
GFPT2	AGPAT4	Co-expression	Dobbin-Giordano-2005
MAN2A1	CBR1	Co-expression	Dobbin-Giordano-2005
CP	CH	Co-expression	Innocenti-Brown-2011
CBR1	NMRK1	Co-expression	Innocenti-Brown-2011
HOGA1	CBR1	Co-expression	Innocenti-Brown-2011
NQO2	HOGA1	Co-expression	Innocenti-Brown-2011
GCKR	CP	Co-expression	Innocenti-Brown-2011
MAN2A1	CP	Co-expression	Innocenti-Brown-2011
UGT2B11	UGT2B10	Co-expression	Innocenti-Brown-2011
HEPH	GSTM5	Co-expression	Innocenti-Brown-2011
UGT2B17	UGT2B10	Co-expression	Innocenti-Brown-2011
UGT2B17	UGT2B11	Co-expression	Innocenti-Brown-2011
LCLAT1	CBR1	Co-expression	Innocenti-Brown-2011
UGT2B15	UGT2B10	Co-expression	Innocenti-Brown-2011
UGT2B15	UGT2B11	Co-expression	Innocenti-Brown-2011
UGT2B15	UGT2B17	Co-expression	Innocenti-Brown-2011
CBR1	BDH2	Co-expression	Noble-Diehl-2008
HOGA1	PHKG1	Co-expression	Noble-Diehl-2008
UGT2B11	UGT2B10	Co-expression	Noble-Diehl-2008
UGT2B17	UGT2B10	Co-expression	Noble-Diehl-2008
UGT2B17	UGT2B11	Co-expression	Noble-Diehl-2008
UGT2B15	UGT2B10	Co-expression	Noble-Diehl-2008
UGT2B15	UGT2B11	Co-expression	Noble-Diehl-2008
UGT2B15	UGT2B17	Co-expression	Noble-Diehl-2008
CBR1	NQO1	Co-expression	Ramaswamy-Golub-2001
GGT7	CA11	Co-expression	Ramaswamy-Golub-2001
CBR1	COX11	Co-expression	Chen-Brown-2002
NQO2	GSTM5	Co-expression	Chen-Brown-2002
NQO2	CBR1	Co-expression	Chen-Brown-2002
GCKR	CBR1	Co-expression	Chen-Brown-2002
UGT2B15	GCKR	Co-expression	Chen-Brown-2002
CP	PHKG1	Co-expression	Burington-Shaughnessy-2008
GFPT2	CP	Co-expression	Burington-Shaughnessy-2008
HEPH	CP	Co-expression	Burington-Shaughnessy-2008
UGT2B15	UGT2B17	Co-expression	Burington-Shaughnessy-2008
AGPAT4	MAN2A2	Co-expression	Roth-Zlotnik-2006
CBR1	AGPAT4	Co-expression	Roth-Zlotnik-2006
HOGA1	BDH2	Co-expression	Roth-Zlotnik-2006
NQO2	HOGA1	Co-expression	Roth-Zlotnik-2006
MAN2A1	AGPAT4	Co-expression	Roth-Zlotnik-2006
MAN2A1	CBR1	Co-expression	Roth-Zlotnik-2006
UGT2B15	GCKR	Co-expression	Roth-Zlotnik-2006
CP	PHKG1	Co-expression	Rieger-Chu-2004

MAN2B2	PHKG1	Co-expression	Rieger-Chu-2004
UGT2B17	UGT2B11	Co-expression	Rieger-Chu-2004
UGT2B15	UGT2B11	Co-expression	Rieger-Chu-2004
UGT2B15	UGT2B17	Co-expression	Rieger-Chu-2004
CP	CH	Co-expression	Mallon-McKay-2013
CYC1	CYCS	Co-expression	Mallon-McKay-2013
CYC1	CBR1	Co-expression	Mallon-McKay-2013
UGT2B11	UGT2B10	Co-expression	Mallon-McKay-2013
HEPH	AGPAT4	Co-expression	Mallon-McKay-2013
UGT2B17	NOX1	Co-expression	Mallon-McKay-2013
UGT2B15	NOX1	Co-expression	Mallon-McKay-2013
UGT2B15	UGT2B17	Co-expression	Mallon-McKay-2013
CBR1	CP	Co-localization	Schadt-Shoemaker-2004
UGT2B11	UGT2B10	Co-localization	Schadt-Shoemaker-2004
UGT2B15	UGT2B10	Co-localization	Schadt-Shoemaker-2004
UGT2B15	UGT2B11	Co-localization	Schadt-Shoemaker-2004
AGPAT4	MAN2A2	Co-localization	Johnson-Shoemaker-2003
AGPAT4	HS3ST3A1	Co-localization	Johnson-Shoemaker-2003
GCKR	UGT2B10	Co-localization	Johnson-Shoemaker-2003
GFPT2	SULT1C4	Co-localization	Johnson-Shoemaker-2003
MAN2A1	AGPAT4	Co-localization	Johnson-Shoemaker-2003
HS3ST3A1	SULT1C4	Shared protein domains	INTERPRO
GFPT1	GGTLC1	Shared protein domains	INTERPRO
CBR1	BDH2	Shared protein domains	INTERPRO
HOGA1	NPL	Shared protein domains	INTERPRO
CYC1	CYCS	Shared protein domains	INTERPRO
NQO2	NQO1	Shared protein domains	INTERPRO
GCKR	GFPT1	Shared protein domains	INTERPRO
HEPHL1	CP	Shared protein domains	INTERPRO
GFPT2	GGTLC1	Shared protein domains	INTERPRO
GFPT2	GFPT1	Shared protein domains	INTERPRO
GFPT2	GCKR	Shared protein domains	INTERPRO
MAN2A1	MAN2A2	Shared protein domains	INTERPRO
UGT2B11	UGT2B10	Shared protein domains	INTERPRO
HEPH	CP	Shared protein domains	INTERPRO
HEPH	HEPHL1	Shared protein domains	INTERPRO
MAN2B2	MAN2A2	Shared protein domains	INTERPRO
MAN2B2	MAN2A1	Shared protein domains	INTERPRO
GGTLC2	GGTLC1	Shared protein domains	INTERPRO
GGTLC2	GFPT1	Shared protein domains	INTERPRO
GGTLC2	GFPT2	Shared protein domains	INTERPRO
GGT2	GGTLC1	Shared protein domains	INTERPRO
GGT2	GFPT1	Shared protein domains	INTERPRO
GGT2	GFPT2	Shared protein domains	INTERPRO
GGT2	GGTLC2	Shared protein domains	INTERPRO
GGTLC3	GGTLC1	Shared protein domains	INTERPRO
GGTLC3	GFPT1	Shared protein domains	INTERPRO
GGTLC3	GFPT2	Shared protein domains	INTERPRO
GGTLC3	GGTLC2	Shared protein domains	INTERPRO

GGTLC3	GGT2	Shared protein domains	INTERPRO
MAN2C1	MAN2A2	Shared protein domains	INTERPRO
MAN2C1	MAN2A1	Shared protein domains	INTERPRO
MAN2C1	MAN2B2	Shared protein domains	INTERPRO
UGT2B17	UGT2B10	Shared protein domains	INTERPRO
UGT2B17	UGT2B11	Shared protein domains	INTERPRO
LCLAT1	AGPAT4	Shared protein domains	INTERPRO
UGT2B15	UGT2B10	Shared protein domains	INTERPRO
UGT2B15	UGT2B11	Shared protein domains	INTERPRO
UGT2B15	UGT2B17	Shared protein domains	INTERPRO
GGT6	GGTLC1	Shared protein domains	INTERPRO
GGT6	GFPT1	Shared protein domains	INTERPRO
GGT6	GFPT2	Shared protein domains	INTERPRO
GGT6	GGTLC2	Shared protein domains	INTERPRO
GGT6	GGT2	Shared protein domains	INTERPRO
GGT6	GGTLC3	Shared protein domains	INTERPRO
GGT7	GGTLC1	Shared protein domains	INTERPRO
GGT7	GFPT1	Shared protein domains	INTERPRO
GGT7	GFPT2	Shared protein domains	INTERPRO
GGT7	GGTLC2	Shared protein domains	INTERPRO
GGT7	GGT2	Shared protein domains	INTERPRO
GGT7	GGTLC3	Shared protein domains	INTERPRO
GGT7	GGT6	Shared protein domains	INTERPRO
CTD-2192J16.22	MAN2A2	Shared protein domains	INTERPRO
CTD-2192J16.22	MAN2A1	Shared protein domains	INTERPRO
CTD-2192J16.22	MAN2B2	Shared protein domains	INTERPRO
CTD-2192J16.22	MAN2C1	Shared protein domains	INTERPRO

Supplementary Table S4:

Acronyms of genes involved in regulation of nucleic acid metabolism, metabolism and energy pathways.

Regulation of nucleobase, nucleoside, nucleotide and nucleic acid metabolism	Metabolism	Energy Pathways
HNF1 homeobox A (HNF1A)	UDP glucuronosyltransferase family 2 member B10 (UGT2B10)	UDP glucuronosyltransferase family 2 member B10 (UGT2B10)
zinc finger and SCAN domain containing 4 (ZSCAN4)	carbonic anhydrase 11 (CA11)	carbonic anhydrase 11 (CA11)
elongation factor for RNA polymerase II 2 (ELL2)	heparan sulfate-glucosamine 3-sulfotransferase 3A1 (HS3ST3A1)	heparan sulfate-glucosamine 3-sulfotransferase 3A1 (HS3ST3A1)

zinc finger protein 45 (ZNF45)	carbonyl reductase 1 (CBR1)	carbonyl reductase 1 (CBR1)
FMR1 interacting protein 2 (NUFIP2)	NAD(P)H quinone dehydrogenase 1 (NQO1)	NAD(P)H quinone dehydrogenase 1 (NQO1)
RALY RNA binding protein-like (RALYL)	Rab escort protein 1 (CHM)	Rab escort protein 1 (CHM)
zinc finger protein 280D (ZNF280D)	1-acylglycerol-3-phosphate O-acyltransferase 4 (AGPAT4)	n icotinamide Riboside Kinase (NMRK)
nuclear transcription factor Y subunit alpha (NFYA)	nicotinamide riboside kinase 1 (NMRK1)	3-hydroxybutyrate dehydrogenase, type 2 (BDH2)
jade family PHD finger 2 (JADE2)	3-hydroxybutyrate dehydrogenase, type 2 (BDH2)	phosphorylase kinase catalytic subunit gamma 1 (PHKG1)
nuclear receptor coactivator 2 (NCOA2)	phosphorylase kinase catalytic subunit gamma 1 (PHKG1)	glutamine--fructose-6-phosphate transaminase 1 (GFPT1)
zinc finger protein 214 (ZNF214)	glutamine--fructose-6-phosphate transaminase 1 (GFPT1)	COX11 cytochrome c oxidase copper chaperone (COX11)
zinc finger protein 222 (ZNF222)	COX11 cytochrome c oxidase copper chaperone (COX11)	ceruloplasmin (CP)
zinc finger CCHC-type containing 11 (ZCCHC11)	ceruloplasmin (CP)	NADPH oxidase 1 (NOX1)
PR domain 2 (PRDM2)	NADPH oxidase 1 (NOX1)	glutathione S-transferase mu 5 (GSTM5)
zinc finger protein 148 (ZNF148)	glutathione S-transferase mu 5 (GSTM5)	RNA guanylyltransferase and 5'-phosphatase (RNGTT)
poly(rC) binding protein 3 (PCBP3)	RNA guanylyltransferase and 5'-phosphatase (RNGTT)	gamma-glutamyltransferase light chain 1 (GGTLC1)
ERCC excision repair 4, endonuclease catalytic subunit (ERCC4)	gamma-glutamyltransferase light chain 1 (GGTLC1)	sulfotransferase family 1C member 4 (SULT1C4)
mediator complex subunit 17 (MED17)	sulfotransferase family 1C member 4 (SULT1C4)	cytochrome c, somatic (CYCS)
RB associated KRAB zinc finger (RBAK)	cytochrome c, somatic (CYCS)	mannosidase alpha class 2A member 2 (MAN2A2)
single-minded family bHLH transcription factor 2 (SIM2)	mannosidase alpha class 2A member 2 (MAN2A2)	N-acetylneuraminate pyruvate lyase (NPL)
Sp7 transcription factor (SP7)	N-acetylneuraminate pyruvate lyase (NPL)	steroid 5 alpha-reductase 1 (SRD5A1)
ring finger and CCCH-type domains 2 (RC3H2)	steroid 5 alpha-reductase 1 (SRD5A1)	abhydrolase domain containing 1 (ABHD1)
ALX homeobox 4 (ALX4)	abhydrolase domain containing 1 (ABHD1)	
homeobox C13 (HOXC13)		
autoimmune regulato (AIRE)		

small EDRK-rich factor 2 (SERF2)		
transcription elongation factor B subunit 3 (TCEB3)		
DEAD-box helicase 46 (DDX46)		
Sin3A associated protein 18 (SAP18)		
LIM domain 7 (LMO7)		
zinc finger protein 438 (ZNF438)		
zinc finger protein 566 (ZNF566)		
forkhead box Q1 (FOXQ1)		
zinc finger protein 568 (ZNF568)		
histone deacetylase 4 (HDAC4)		
ribonuclease P/MRP subunit p30 (RPP30)		

Supplementary Table S5:

Interactions of genes involved in metabolism in 10nM BPA treated ST-BeWos

Gene 1	Gene 2	Interaction	Network
SEC61G	WDR44	Co-expression	Noble-Diehl-2008
L1TD1	WDR44	Co-expression	Noble-Diehl-2008
L1TD1	SEC61G	Co-expression	Noble-Diehl-2008
NOP10	WDR44	Co-expression	Noble-Diehl-2008
NOP10	SEC61G	Co-expression	Noble-Diehl-2008
SGPP1	WDR44	Co-expression	Noble-Diehl-2008
SGPP1	SEC61G	Co-expression	Noble-Diehl-2008
SGPP1	NOP10	Co-expression	Noble-Diehl-2008
ZYG11B	SLC20A2	Co-expression	Noble-Diehl-2008
MARS	XPO6	Co-expression	Noble-Diehl-2008
PIR	WDR44	Co-expression	Noble-Diehl-2008
PIR	SEC61G	Co-expression	Noble-Diehl-2008
PEAR1	SLC6A6	Co-expression	Noble-Diehl-2008
CLCN4	MIP	Co-expression	Noble-Diehl-2008
SYT10	SCN5A	Co-expression	Noble-Diehl-2008
PRY2	SLC6A6	Co-expression	Noble-Diehl-2008
C12orf73	SLC25A40	Co-expression	Noble-Diehl-2008
SHFM1	WDR44	Co-expression	Noble-Diehl-2008
SHFM1	SEC61G	Co-expression	Noble-Diehl-2008
SHFM1	NOP10	Co-expression	Noble-Diehl-2008
TRIM36	SLC20A2	Co-expression	Noble-Diehl-2008
ZBTB9	XPO6	Co-expression	Noble-Diehl-2008

CARD16	WDR44	Co-expression	Noble-Diehl-2008
FABP5	SEC61G	Co-expression	Burington-Shaughnessy-2008
TRAM1	SLC20A2	Co-expression	Burington-Shaughnessy-2008
TRAM1	SEC61G	Co-expression	Burington-Shaughnessy-2008
WISP1	SLC6A6	Co-expression	Burington-Shaughnessy-2008
CLCN4	RRBP1	Co-expression	Burington-Shaughnessy-2008
CLCN4	PIR	Co-expression	Burington-Shaughnessy-2008
LTK	SCN5A	Co-expression	Burington-Shaughnessy-2008
LTK	CACNA2D2	Co-expression	Burington-Shaughnessy-2008
GNGT1	SCN5A	Co-expression	Burington-Shaughnessy-2008
GNGT1	CACNA2D2	Co-expression	Burington-Shaughnessy-2008
CNTF	SLC6A6	Co-expression	Burington-Shaughnessy-2008
COG4	XPO6	Co-expression	Rieger-Chu-2004
TRAM1	SEC61G	Co-expression	Rieger-Chu-2004
CLCN4	XPO6	Co-expression	Rieger-Chu-2004
